# Lambda Phage-Based Antibody-Stimulating Platform Targeting EGFRvIII

**DOI:** 10.3390/vaccines14030282

**Published:** 2026-03-23

**Authors:** Meredith Bush, Manoj Rajaure, Calla Gentilucci, Phuoc Le, Xintian Li, Sankar Adhya

**Affiliations:** 1 Laboratory of Molecular Biology, Center for Cancer Research, National Cancer Institute, National Institutes of Health, Bethesda, MD 20892, USA; mail2mrajaure@gmail.com (M.R.); callayuna@icloud.com (C.G.); phuocle@mail.nih.gov (P.L.); xintian.li@nih.gov (X.L.); 2 Laboratory of Immunology, National Eye Institute, National Institutes of Health, 10 Center Drive, Bethesda, MD 20892, USA

**Keywords:** phage display, lambda, EGFRvIII, immunogenicity, immunotherapy, vector, antibody, peptide, cancer

## Abstract

Background/Objectives: Bacteriophage-based display has been utilized for a variety of purposes, such as to assemble protein libraries and conduct biopanning. We have created a modified lambda (λ) bacteriophage platform, ideal for the display and delivery of proteins. Our system utilizes counter-selection recombineering for versatile modification, temperature-sensitive induction for timely lysate production, and an arabinose-inducible mechanism for high-titer, stable yield. Here, we investigated the ability of this specialized λ phage display platform to stimulate highly specific antibodies in mice against the displayed cancer-variant cell-surface receptor EGFRvIII, demonstrating its potential in cancer immunotherapy and broader vaccine development. Methods: λ display immunogenicity was explored by generating fusion proteins between the λ head protein D and a 13-mer peptide from the N terminus of glioblastoma variant cell-surface receptor, EGFRvIII. The 13-mer peptide was fused to either the N or C terminus of the λD protein while λ remained a dormant lysogen in the bacterial host chromosome. Recombinant phage lysates were then generated with ~420 displayed fusion proteins per phage particle. Mice were injected with purified recombinant λ phage without an adjuvant via both intraperitoneal and intramuscular routes, and sera harvested at various timepoints were profiled for immunogenicity. Results: Analysis of serum samples by ELISA and Western blotting demonstrated the ability of the λD~EGFRvIII phage display, especially in the C-terminal fusion construction, to elicit a robust anti-EGFRvIII humoral response by either injection route. Notably, the antibody response was highly specific to EGFRvIII without exhibiting cross-reactivity to wild-type EGFR. Conclusions: The data generated in this study demonstrate the λ system’s immunotherapeutic potential as a high-titer, stable, self-adjuvanting vector for the stimulation of robust antibody titers with defined specificity.

## 1. Introduction

Early foundational work established the *Escherichia coli* (*E. coli*) lambda (λ) bacteriophage as a platform for high-density antigen display [1,2]. We have previously described a modified λ vector for the display of foreign peptides and proteins on the λD major capsid protein, which may be employed for a variety of applications [3]. Here, we use λ to deliver antigenic proteins to a murine mammalian system for the generation of a highly specific immune response, investigating the utility of our λ display vector for cancer immunotherapy. In contrast to “passive” immunotherapy approaches that deliver targeted antibodies for rapid, high-specificity effects, such as hybridoma-derived monoclonal antibody treatment, an in vivo, “active” phage-based vaccine can elicit a comprehensive endogenous immune response [4,5,6]. Phage display systems hold broad potential to overcome tumor immunosuppression while simultaneously promoting antigen-specific targeting [7,8].

A noteworthy λ phage feature, especially in its use as an antibody-stimulating platform, is the phage’s ability to act as an adjuvant, provoking a host’s immune system without chemical stimulants [9,10,11]. Additionally, the multivalent nature of the λ display system, presenting hundreds of antigen copies on the geometrically rigid capsid of each particle, aids in the stimulation of a robust immune response [12,13,14,15]. Several studies have reported increased immunogenicity using phage-based vaccination compared to other vaccine platforms, such as recombinant protein vaccines [16] and naked DNA vaccines [17]. Other advantages of the λ display system include the simple, inexpensive, and rapid production of protein displays [7]; long-term stability at ambient temperatures [18]; and an excellent safety profile due to λ’s natural ubiquity and inability to infect eukaryotic cells [8,19]. The λ platform described here functions as an adaptable delivery vector and adjuvant capable of driving an immune response against a defined passenger antigen.

For use in cancer immunotherapy, phage-based antigen delivery platforms offer distinct advantages due to their ability to interact with both the innate and adaptive immune system, stimulating multi-pronged anti-cancer responses [7]. When entering the mammalian system, bacteriophages trigger the innate immune system via pattern recognition receptors, such as Toll-like receptors (TLRs), which detect phage motifs and activate non-specific cytokine production [20,21]. This response can help shift the immunosuppressive tumor microenvironment toward an immunostimulatory state [7]. Upon recognition of the phage as a viral particle, phage virions are phagocytized and broken down by antigen-presenting cells (APCs), initiating adaptive immune system responses [22,23]. The processed proteins, including phage-displayed antigens, are then presented through two major histocompatibility complex (MHC) pathways, MHC Class I and MHC Class II [24,25]. These pathways initiate either CD8+ cytotoxic T lymphocyte activation targeting the specified antigen (MHC-I) [26], or CD4+ T helper cell stimulation triggering antibody-mediated destruction of a cell presenting the specified antigen (MHC-II) [27]. Phage-based systems present the opportunity to directly deliver a high copy number antigen in high-titer doses while simultaneously stimulating multiple immune pathways.

The body of research investigating phage display vaccines for cancer immunotherapy is continuously growing, reporting the stimulation of humoral and anti-tumor activity. Noteworthy studies displaying peptides derived from HER2 on gpD of λ phage nanoparticles have demonstrated CD8+ cytotoxic T cell responses and therapeutic anti-tumor effects in BALB/c breast tumor mouse models [11,28,29]. Targeting hepatocellular carcinoma (HCC), a study utilizing λ phage-displayed ASPH peptides found antigen-specific CD4+/CD8+ lymphocyte production, Th1 and Th2 cytokine secretion, and inhibited growth in a liver HCC tumor mouse model [10]. Beyond λ phage display, virus-like particles derived from Qβ phage [30] and T4 phage vaccines [31] have generated specific humoral responses to displayed tumor-associated proteins. These studies highlight the need for further investigation into these innovative approaches to cancer treatment.

The cancer-associated antigen selected for λD display immune stimulation in this study was derived from the N terminus of extracellular EGFRvIII, a mutant form of epidermal growth factor receptor (EGFR) found in 30% of glioblastoma multiforme [32]. Upon ligand binding, wild-type EGFR regulates an essential signaling system that controls cell growth, proliferation, apoptosis, angiogenesis, migration, and adhesion [33,34,35]. The EGFRvIII mutation is the product of an 801-base pair in-frame deletion, which eliminates EGFR’s extracellular domain ligand-binding residues. As a result, EGFRvIII is unable to bind ligands but remains constitutively active, driving low-level activation of downstream cell growth signaling pathways [32,36]. This tumor-specific EGFRvIII mutation aids tumor growth, confers resistance to radiation and chemotherapy, and is not found in normal epithelial cells [37]. A feature which makes EGFRvIII ideal for targeted immunotherapy is the extracellular N terminus, where a novel glycine is created in the deletion junction between amino acids 6-273 [38]. The first thirteen amino acids of the N terminus, including the novel junction, can therefore be used as an antigen specific to the mutated receptor found only on the surface of cancer cells. This 13-mer peptide, called rindopepimut when chemically synthesized with an additional C-terminal cysteine and conjugated to keyhole limpet hemocyanin (KLH) [39], has been tested in several glioblastoma immunotherapy clinical trials [36,38,40,41,42]. Though the synthesized peptide therapeutic ultimately did not improve outcomes in glioblastoma patients in phase III clinical trials [43], we chose the cancer-specific EGFRvIII 13-mer peptide to assess the potential of the λ vector platform to drive a specific and robust anti-tumor immune response.

In a study by Fidanza et al. (2021) [44], subcutaneously injecting rindopepimut with a tyrosine at the novel junction (Y-version) instead of glycine (G-version) increased overall survival and decreased tumor volume in a mouse subcutaneous tumor model as well as in an intracranial glioma model. The study credited the survival advantage to enhanced proteasomal processing of the Y-version antigen. Since the modified Y-version EGFRvIII antigen may be clinically useful, we evaluated its ability to elicit antibody responses in mice when displayed on λ compared to the naturally occurring G-version displayed antigen.

## 2. Materials and Methods

The following phage methodologies were found and adapted from Arber et al. [45] (pp. 433–466) and Georgopoulos C. et al. [46] (pp. 279–304) in *Lambda II*, edited by Hendricks et al. The details of strain construction are adapted from Thomason et al. 2023 [47]. All platform development and induction protocol details are described further in Bush et al. 2026 [3].

### 2.1. Study Design

**Figure 1:**
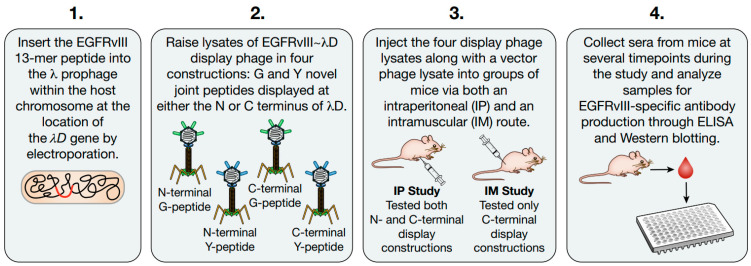
EGFRvIII display phage antibody stimulation study design schematic. (**1**) λ prophage are modified as lysogens within the host chromosome by electroporating DNA of the display peptide flanked by homologous arms. (**2**) After verification of successful incorporation of intended display proteins to the N or C terminus of λD, prophages are induced to generate display phage lysates. (**3**) Display phage and vector phage lysates were purified and injected into groups of mice in two distinct studies through either an intraperitoneal (IP) or intramuscular route (IM). IP study mice received both N- and C-terminal EGFRvIII peptide display constructions. IM study mice receive only C-terminal peptide display phage. (**4**) Sera were harvested from mice at multiple timepoints throughout both studies until the terminal bleed. Sera were then analyzed for EGFRvIII-specific antibody production through ELISA analysis and Western blotting.

This study has several aims: to provide data on the novel λ display platform’s ability to generate antibodies in a mammalian system, to determine the optimal construction for antigen display, and to determine the specificity of the antibodies produced. We designed four versions of the EGFRvIII~λD phage display fusion: a Y or G novel joint antigen fused to either the N or C terminus of λD (Figure 1). We engineered these prophage constructs through counter-selection recombineering, verified sequence accuracy, and raised lysates of the display strains through temperature-mediated induction. We then injected the purified display and control phage into BALB/c mice via an intraperitoneal route (IP) and an intramuscular route (IM). The resulting sera were analyzed by ELISA and Western blotting to quantify the anti-EGFRvIII humoral response produced by display phage injection.

### 2.2. The λ Display Platform

The *E. coli* host and λ prophage strains used to generate the display phages for this study were each engineered in several ways to simplify the display process and improve phage yield (Table 1 and Table 2). The *cI857* mutation allows for temperature-sensitive induction of the lytic pathway for engineering simplification [48]; the *Sam7* mutation inactivates the phage holin protein to maximize phage yield during induction [49]; and the long-circulating *E* mutation increases phage circulation in mammalian blood [50]. Deletions of non-essential genes allow for the accommodation of larger fusion proteins [51], while several other mutations have been employed to streamline the use of λ as a display platform [3].

The λ vector platform described here utilizes gpD (gene product D) for protein display, which assembles in trimers on the phage head at a high copy number of ~420 per phage particle [46,52] (pp. 279–304). Both the λD amino and carboxyl termini protrude outward from the virion, allowing for fusion proteins to join either end for display [53,54]. While fusions of foreign proteins to the λD protein C terminus are not known to interact with the coordinately expressed major capsid protein gpE, there is some evidence that fusions to the λD N terminus may interact with gpE and influence particle stability [19,53,55]. Our study investigates the virion stability and immunogenic potential of the λ vector displaying antigens at either terminus of the λD protein.

Strain SJ_XTL175 [56], a wild-type MG1655 derivative, was used as the starting strain and modified with the above mutations and many others to create strain XTL1026 [3]. When modified with the counter-selectable *tetA-sacB* marker cassette [57] inserted just upstream of the *D* gene in the prophage, the resulting strain, XTL1059, can be easily recombined with the fusion protein at the N terminus of *λD*. Strain XTL1059 was used to create the N-terminal EGFRvIII~λD fusion phage described in this study (MS41 and MS42). Strain CC001, used for the C-terminal fusion strains in this study (MS45 and MS48), is a modification of XTL1026 where a *ccdB-kan* cassette marker is inserted downstream of *λD* to be exchanged with the linker and antigen sequence at the C terminus of *λD*. This alternative counter-selection method was used to avoid sucrose-resistant non-recombinants that require extra screening when using the *tetA-sacB* cassette. A four-amino acid linker sequence was used in triplicate between the *λD* gene and the antigenic peptide to create *λD* fusions (GlyGlyGlySer) (Adapted from Brinkmann, Lee, and Pastan, 1993) [58].

One important modification featured in XTL1026 and its derivatives used here is the placement of *D* under *AraC* regulation and arabinose-inducible control in the host chromosome. This allows wild-type λD protein expression level to be tuned in every cell in response to the extracellular arabinose concentration [56]. This mechanism was used here to induce a supply of wild-type λD during phage assembly, preventing potential instability caused by the fusion and promoting a high-titer yield.

### 2.3. Prophage Induction and Phage Preparation for Animal Studies

Induction: Prophage *λD* genetic fusions were confirmed by PCR amplification and sequencing. Strains MS41, MS42, MS45, and MS48 were chosen from among ten recombinant strains due to sequencing results demonstrating accurate fusion of the EGFRvIII peptide with a linker to the intended *λD* terminus. After verification, fusion prophages were then induced to create lysates. The *E. coli* strain with a recombinant prophage was grown at 32 °C until OD600 0.25, at which time the temperature was raised to 42 °C to inactivate the CI temperature-sensitive repressor. After temperature induction, the phage became lytic but did not lyse the culture due to the *Sam7* mutation, which inactivated the holin protein needed for escape from the cell. To lyse by the freeze/thaw method, the resuspended cell pellet was frozen at −80 °C overnight and thawed the next day at 37 °C. The resultant crude lysate was spun down to remove bacterial debris before filtration of the supernatant lysate. The lysate was then titered by spotting 5 μL 10-fold serial dilutions on the *supF* host strain XTL1212. The titer (plaque forming unit/mL) was determined by calculating the number of plaques formed, divided by the volume spotted in mL, multiplied by the dilution factor.

Tangential flow filtration and endotoxin assay: The lysates were filtered for removal of endotoxins and other debris by passage through a Pellicon^®^ Mini Cassette 0.1 mm Composite Regenerated Cellulose filter from MilliporeSigma, Burlington, MA, USA. The phage buffer used in tangential flow filtration was composed of 10 mM Tris and 10 mM MgCl_2_, which allowed for long-term stability [18]. The phage lysates were filtered by passing 7 L of buffer through the filter system until the sample became clear and collecting the purified sample in a small volume (5–15 mL) of phage buffer. Endotoxin readings of the engineered fusion phage lysates after filtration were taken by Endosafe^®^ Nextgen-PTS™ (Portable Test System) from Charles River, Wilmington, MA, USA to ensure safe endotoxin levels for use in the mouse experiments.

### 2.4. Mouse Experiments

We performed two live mouse studies, injecting the fusion and control phage lysates into BALB/c female mice and harvesting sera throughout the study. Both mouse studies were performed under Integrated Biotherapeutics (Rockville, MD, USA) Institutional Animal Care and Use Committee (IACUC) protocol #160805, approved 11 September 2022. The animal studies were conducted in compliance with the Public Health Service and NIH policies and the Guide for the Care and Use of Laboratory Animals. Integrated Biotherapeutic’s Animal Welfare Assurance was approved by the Office of Laboratory Animal Welfare (Assurance No. D17-00974).

Mice were housed in Innovive individually ventilated disposable cages (5 mice/cage) under controlled temperature, humidity, and lighting conditions, with ad libitum access to irradiated feed and sterile drinking water. All cages were changed at least once per week. Animals were monitored at least twice daily by trained staff. All procedures were performed by Integrated Biotherapeutics technicians trained by staff veterinarians and conducted in accordance with approved DOPs.

Animals were humanely euthanized by cardiac puncture according to approved IACUC protocol #160805 to obtain the endpoint terminal bleeds. Secondary confirmation of death was performed by cervical dislocation and exsanguination.

#### 2.4.1. IP Study

Thirty BALB/c female mice were immunized intraperitoneally in groups of five with λD~EGFRvIII fusion phages, vector phage, or phage buffer. Injections consisted of 500 μL of 2 × 10^9^ total phage particles in 10 mM Tris and 10 mM MgCl_2_ phage buffer. Mice were injected on Days 0, 13, and 34. Serum was collected from each mouse on Days 0, 13, 27, and 49. The final bleed was terminal. Injection groups were as follows: N-Terminal (G-version peptide) Phage (YMS41), N-Terminal (Y-version peptide) Phage (YMS42), C-Terminal (G-version peptide) Phage (YMS45), C-Terminal (Y-version peptide) Phage (YMS48), Vector λ Phage (YXTL1026), and 10 mM Tris/10 mM MgCl_2_ phage Buffer.

#### 2.4.2. IM Study

Eighteen BALB/c female mice were immunized three times intramuscularly with λD~EGFRvIII C-terminal fusion phages, vector phage, or 1× PBS buffer. Injection groups consisted of five mice for phage injections and three mice in the buffer injection group. Phage samples were diluted 10× in 1× PBS directly before injection to decrease magnesium content of phage lysate buffer for optimal mouse health. Injections consisted of 50 μL of 2 × 10^9^ total pfu into the right hind thigh. Mice were immunized on Days 0, 13, and 27. Serum was collected from each mouse on Days 0, 13, 27, and 42. The final bleed was terminal. Injection groups were as follows: C-Terminal (G-version peptide) Phage (YMS45), C-Terminal (Y-version peptide) Phage (YMS48), Vector λ Phage (YXTL1026), and 1× PBS Buffer.

### 2.5. ELISA

EGFRvIII-specific antibodies in mouse sera were assessed by enzyme-linked immunosorbent assay (ELISA). Antigen capture was performed using Thermo Fisher (Waltham, MA, USA) ELISA plates according to manufacturers’ instructions. Two 13-mer synthetic EGFRvIII peptides corresponding to G and Y novel junctions, as well as recombinant His-tagged extracellular domain EGFR and EGFRvIII proteins, were used as capture antigens with plate formats optimized for peptide or His-tag immobilization, respectively.

Synthetic peptides produced by GenScript Inc. (Piscataway, NJ, USA) are the first thirteen amino acids of the EGFRvIII mutant receptor N terminus spanning the deletion junction. EGFRvIII-G (LEEKKGNYVVTDH) is the sequence found in the naturally occurring cancer variant. EGFRvIII-Y (LEEKKYNYVVTDH) replaces the novel joint glycine with a tyrosine for potential added proteasomal cleavage [44]. The purity of these peptides was above 95%. They were resuspended to 1 mg/mL in sterile H_2_O following the solubility criteria provided by GenScript and diluted in 1 M carbonate–bicarbonate buffer to 20 μg/mL in each well for the detection of sera antibodies by ELISA.

Recombinant human wild-type EGFR (His-tagged; cat. no. EGR-H5222; Leu25–Ser645; ~70.5 kDa) and EGFRvIII (His-tagged; cat. no. EGI-H52H4; Leu25–Ser378; ~40.5 kDa) extracellular domain proteins expressed in HEK293 cells were purchased from ACRO Biosystems, Newark, DE, USA.

For the peptide-based ELISAs, Nunc MaxiSorp 96-well plates (Thermo Fisher Cat# 436006) were coated with synthetic EGFRvIII peptides at a concentration of 20 µg/mL in 0.1 M carbonate–bicarbonate buffer (100 µL per well) and incubated overnight at 4 °C. The plates were washed with wash buffer (0.05% Tween-20 in 1× PBS) and subsequently blocked with 1% bovine serum albumin (BSA) in 1× PBS for 2 h at room temperature. Following the removal of the blocking solution, the plates were washed prior to serum addition.

For the protein-based ELISAs, His-tagged extracellular domain EGFR and EGFRvIII proteins were immobilized on Pierce™ Nickel-Coated 96-well plates (Thermo Fisher Cat#15442) by overnight incubation at 4 °C in 1× PBS. The proteins were coated at 20 µg/mL (100 µL per well) in the IP study. In the IM study, the EGFRvIII protein was coated at 20 µg/mL, while the wild-type EGFR protein was coated at 34.8 µg/mL to normalize the molar concentrations between the proteins. After overnight incubation, the plates were washed according to the manufacturer’s instructions, and the serum samples were added directly to the plates without an additional blocking step.

For both peptide- and protein-based assays, mouse sera were diluted and added to plates, followed by incubation for 1 h at room temperature. Plates were then washed three times and incubated for 1 h with horseradish peroxidase (HRP)–conjugated anti-mouse IgG secondary antibody diluted in assay buffer. After three additional washes, 100 µL of TMB substrate was added to each well and developed for 12 min at room temperature. Color development was terminated by addition of 5.7% sulfuric acid stop solution, and absorbance was measured at 450 nm using a microplate reader. Background absorbance measured at 562 nm was subtracted from the 450 nm signal.

IP study sera were tested by ELISA in 2-fold dilutions (1:10, 1:20, 1:40, 1:80, and 1:160), and IM study sera were tested by ELISA in the following dilutions: 1:80, 1:100, 1:300, and 1:1000. ELISA values for both studies are shown for serum dilutions within the linear range of detection, determined for both studies to be 80× dilution, where values were no longer saturated. Each data point represents the mean of two readings per serum. Group values are reported as the mean of five mice per group (N = 5), except for the buffer-injected group in the IM study (N = 3). All figure generation and analyses were performed using GraphPad Prism 10, and comparative analyses between groups were performed using two-tailed nonparametric Mann–Whitney U test, generated in GraphPad Prism 10. This statistical test was chosen as the ideal method for data with high variance and low sample size (N = 5). *P* value asterisks are awarded as follows: ns: *p* > 0.05 (not significant); * 0.01 < *p* ≤ 0.05 (significant); ** 0.001 < *p* ≤ 0.01 (very significant). The highest U value (25) corresponds to *p* value = 0.0079 (**), signifying complete and significant separation of groups. Error bars represent standard deviation (SD), calculated in GraphPad Prism 10 software.

### 2.6. Western Blot

Western blots of mouse sera were performed by running the phage and protein samples on Invitrogen Tris-Glycine 4–20% gels. The proteins were then transferred onto a nitrocellulose membrane in tris-glycine–methanol buffer. The membrane was blocked with 5% milk buffer (10 mL) for 2 h followed by the addition of 10 μL of mouse serum or primary antibody in 10 mL of 5% milk buffer. The membranes were washed with 1*×* TNE buffer before the addition of the secondary anti-mouse or anti-rabbit antibody conjugated to peroxidase. Biorad (Hercules, CA, USA) Clarity Max Western ECL substrate was used to visualize the bands with chemiluminescence.

A custom polyclonal anti-λD capsid protein antibody was generated in mouse by GenScript Corp. using purified λD protein as the immunogen. A custom polyclonal anti-EGFRvIII antibody specific to the G-version novel junction was generated in New Zealand rabbit by GenScript Corp. against the synthetic peptide sequence LEEKKGNYVVTDHC conjugated to KLH. The antibody was diluted 1:1000 in milk buffer for use in the Western blots. A recombinant monoclonal antibody against wild-type EGFR (clone 528; sc-120), raised in mouse, was obtained from Santa Cruz Biotechnology, Inc. (Thermo Fisher Scientific).

The following amounts were loaded in each well per sample: 2.2 μg of EGFRvIII extracellular domain protein (100 μg/mL in 22.5 μL sample), 3.9 μg of EGFR extracellular domain protein (174 μg/mL in 22.5 μL sample), 2 × 10^10^ pfu/mL phage in 22.5 μL sample for mouse serum blots, and 5 × 10^9^ pfu/mL phage in 22.5 μL sample for primary antibody blots.

## 3. Results

To evaluate the immunogenicity of the EGFRvIII peptide displayed on bacteriophage λ, four recombinant phage lysates were generated and purified, comprising N- and C-terminal λD fusions of both the G- and Y-variant EGFRvIII peptides. Mice were immunized intraperitoneally (IP) with each recombinant phage, alongside buffer and vector phage controls. A second confirmation study was conducted in which mice were immunized intramuscularly (IM) with the C-terminal G- and Y-variant fusion phages. The serum samples collected from both studies were analyzed for EGFRvIII-specific antibody responses by ELISA and Western blotting.

### 3.1. C-Terminal λD~Antigen Fusion Is More Immunogenic than N-Terminal Fusion

**Figure 2:**
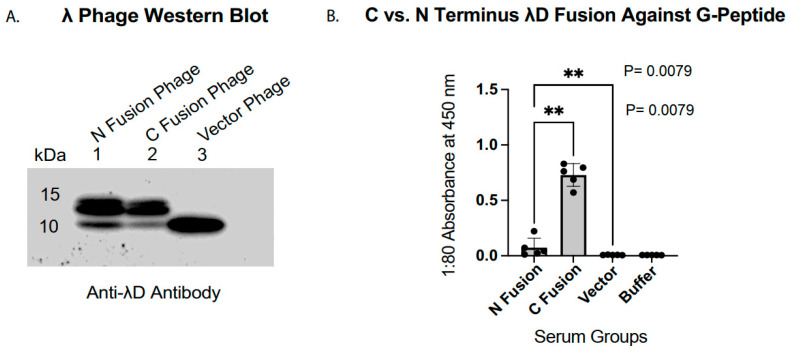
N- and C-terminal λD~antigen fusion validation and immune response. (**A**) Western blot, where G-antigen N-terminal fusion phage (lane 1), G-antigen C-terminal fusion phage (lane 2), and vector phage (lane 3) are loaded onto a gel and blotted with anti-λD antibody. (**B**) ELISA result of final bleed for IP study sera against the synthetic EGFRvIII (G) 13-mer peptide. Each data point is an average of two distinct readings per mouse sera, with five sera averaged per fusion group. The sera tested are from mice injected with N-terminal G-peptide antigen phage, C-terminal G-peptide antigen phage, vector phage, and phage buffer. Mouse sera were tested by ELISA in 2-fold serial dilutions (1:10, 1:20, 1:40, 1:80, and 1:160). A 1:80 dilution was selected for the subsequent analyses, as the corresponding absorbance values fell within the linear range of the assay without evidence of saturation. Mann–Whitney U test statistical analyses and calculations of standard deviation error bars were performed in Prism 10 software. *p* value asterisks are awarded as follows: ** 0.001 < *p* ≤ 0.01 (very significant). Anti-mouse secondary antibody was used in both the Western blot and ELISA assays.

To confirm the successful incorporation of EGFRvIII peptides displayed on the λD capsid protein, purified phage lysates were separated on an SDS-PAGE gel and probed with an anti-λD antibody. The Western blot of G-antigen display phage (Figure 2A) revealed two bands in the 10–15 kDa range in both the N- and C-terminal fusion constructs (lanes 1 and 2). The two recombinant phage upper bands migrated above the wild-type λD band observed in the vector phage control sample (lane 3), consistent with stable fusion of the EGFRvIII peptide to λD. The lower bands, co-migrating with wild-type λD, reflect incorporation of host-encoded λD supplied during phage assembly to ensure recombinant phage stability and subsequent high titer [3,56]. While both the N- and C-terminal display phages incorporated a fraction of wild-type λD, the predominant λD species in the recombinant phage samples corresponded to the fusion protein, confirming efficient antigen display relative to the vector control.

Immunogenicity following IP phage injections demonstrated a pronounced dependence on the fusion construction of the EGFRvIII peptide. ELISA absorbance of sera from mice immunized with G-antigen phage tested against the corresponding G synthetic peptide (Figure 2B) showed substantially higher antibody reactivity in the C-terminal fusion group (mean absorbance 0.73) compared to the N-terminal fusion group (mean absorbance 0.07). Although the N-terminal fusion phage elicited responses above those observed in the vector phage and buffer control groups (*p* = 0.0079), the C-terminal display resulted in a markedly enhanced antibody response. This finding was consistent for each day of the study and was found across a full dilution series for the terminal bleed (Figure S1). Given the low titers observed from the N-terminal display injections, the subsequent IM immunization study was limited to the phage displaying EGFRvIII peptides in the C-terminal λD construction.

### 3.2. Route-Dependent Kinetics of Antibody Responses Following Phage Injection

**Figure 3:**
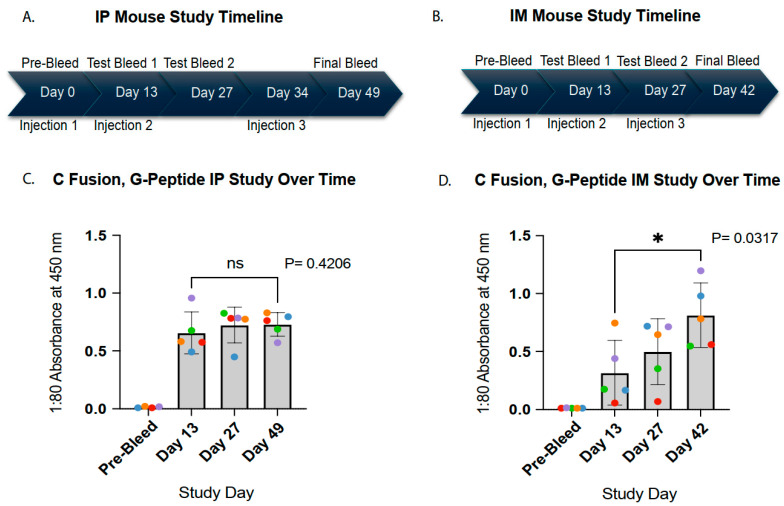
IP and IM injection route immunogenicity trends. Immune response by ELISA of the C-terminal, G-peptide serum group at each bleed timepoint throughout the IP and IM studies against the synthetic EGFRvIII (G) 13-mer peptide. Each data point is an average of two distinct readings per mouse, with five sera averaged per bar and each color representing the same mouse over the course of the study. Anti-mouse secondary antibody was used in the ELISA assays. (**A**) Timeline of IP study bleeds and injections. (**B**) Timeline of IM study bleeds and injections. (**C**) IP study sera antibody titers from the C-terminal, G-peptide group over time. (**D**) IM study sera antibody titers from the C-terminal, G-peptide group over time. Mouse sera were tested by ELISA in the following serial dilutions: IP = 1:10, 1:20, 1:40, 1:80, and 1:160; IM = 1:80, 1:100, 1:300, and 1:1000. A 1:80 dilution was selected for the subsequent analyses for both studies, as the corresponding absorbance values fell within the linear range of the assay without evidence of saturation. Mann–Whitney U test statistical analyses and calculations of standard deviation error bars were performed in Prism 10 software. *p* value asterisks are awarded as follows: ns: *p* > 0.05 (not significant); * 0.01 < *p* ≤ 0.05 (significant).

Administration of identical EGFRvIII~λD C-terminal, G-peptide fusion phage via IP or IM routes resulted in distinct patterns of humoral immune response. Following IP immunization with the G-variant fusion phage in the initial study (Figure 3C), EGFRvIII-specific antibodies measured against the synthetic G-peptide increased rapidly, reaching high levels by two weeks after the primary injection. Antibody titers remained stable following two additional booster doses, with only a slight and non-significant increase above the Day 13 response observed at the final serum collection. This rapid but plateaued response was not evident in the IM study. In contrast, mice injected via the IM route in the second study (Figure 3D) exhibited a gradual, sustained, and significant increase in antibody responses over time.

Longitudinal tracking of individual animals, shown through color-coded data points, further highlight route-dependent differences in the response dynamics. In the IP study, three mice exhibited reduced ELISA absorbance values at the final bleed compared to the preceding timepoint (purple, green, and red), suggesting a stabilization of antibody levels over time. In contrast, all mice in the IM study demonstrated higher antibody signals at the final bleed relative to the prior serum collection, reflecting consistently increasing responses across the cohort. These differing antibody kinetics indicate that IM may be a more favorable injection route for its increasing trend over the plateauing trend of IP.

Individual mouse response variability also differed between the two routes of administration. Antibody responses in the IP study clustered more closely within groups, whereas the IM-immunized mice exhibited greater variability, particularly at the early timepoints, with several animals initially displaying low antibody responses. However, by the final day of serum collection, all IM-immunized mice reached pronounced antibody levels despite continued individual variation. Importantly, group-averaged antibody titers at the terminal timepoint were comparable between the two studies (mean absorbance of 0.73 for IP and 0.81 for IM). While IP and IM immunization produced distinct kinetic profiles of antibody induction, the EGFRvIII~λD fusion phage injections generated comparably strong antibody responses via both routes.

### 3.3. G- and Y-Raised Antibody Specificity to Antigen Peptide Sequence

**Figure 4:**
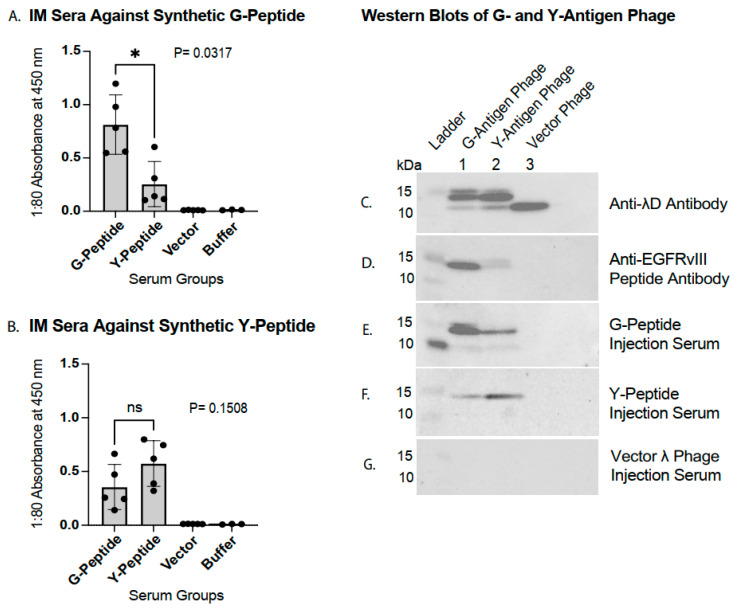
Novel joint amino acid specificity by ELISA and Western blot. ELISA and Western blot antibody detection results are shown for IM study mouse sera from the terminal bleed (Day 42). ELISA absorbance was read at 450 nm. Mann–Whitney U test statistical analyses and calculations of standard deviation error bars were performed in Prism 10 software. *p* value asterisks are awarded as follows: ns: *p* > 0.05 (not significant); * 0.01 < *p* ≤ 0.05 (significant). Each data point is an average of two distinct readings per mouse, with five sera averaged per bar. Mouse sera were tested by ELISA in the following serial dilutions: 1:80, 1:100, 1:300, and 1:1000. A 1:80 dilution was selected for the subsequent analyses, as the corresponding absorbance values fell within the linear range of the assay without evidence of saturation. The assays utilized anti-mouse secondary antibody. (**A**) ELISA of G- and Y-peptide C-terminal display phage, vector phage, and buffer injection sera response against the synthetic EGFRvIII (G) 13-mer peptide. (**B**) ELISA of G- and Y-peptide C-terminal display phage, vector phage, and buffer injection sera response against the synthetic EGFRvIII (Y) 13-mer peptide. (C-G) Western blots, where C-terminal fusion phage with the G-version antigen (lane 1), Y-version antigen (lane 2), and vector phage (lane 3) were loaded onto a gel. Phage samples immobilized on nitrocellulose membranes were then blotted with an anti-λD antibody (**C**), an anti-EGFRvIII peptide antibody (**D**), C-terminal G-peptide fusion phage-injected serum (**E**), C-terminal Y-peptide fusion phage-injected serum (**F**), and vector λ phage-injected serum (**G**). The serum samples with the highest ELISA titers per group were chosen for use in the Western blots. Anti-mouse secondary antibody was used for all blots, except when using the anti-EGFRvIII peptide antibody (**D**), which required anti-rabbit secondary antibody.

To assess G- and Y-antigen immunogenicity, groups of five mice were immunized via an IM route with λ phage displaying either the G- or Y-version EGFRvIII peptide in the C-terminal λD construction. The sera collected at the terminal timepoint were analyzed by ELISA. When tested for binding to the synthetic G-peptide (Figure 4A; corresponding IP study data in Figure S2A), the sera from both injection groups produced signals well above those observed in the vector phage and buffer control groups. However, the mice immunized with the G-version display phage exhibited substantially higher antibody reactivity compared to the Y-version group, with mean absorbance values of 0.81 and 0.25, respectively.

Reciprocal binding specificity was evaluated by analyzing sera from both injection groups against the synthetic Y-peptide (Figure 4B and Figure S2B–D). Sera from the Y-antigen-immunized mice demonstrated a higher mean antibody reactivity to the Y-peptide than sera from the G-version-immunized mice, although this difference was non-significant. However, the IP study, with closer data point groupings, found a significant elevation in the Y-antigen injection group over the G-antigen phage group (Figure S2B). Taken together, the findings from Figure 4A,B and Figure S2 indicate that phage-displayed EGFRvIII antigens elicit antibody responses with preferential binding to the peptide sequence used for immunization and decreased cross-reactivity to peptides differing by a single amino acid.

Antigen specificity was further validated by a Western blot analysis of G-version display phage, Y-version display phage, and vector phage probed with defined primary antibodies (Figure 4C,D). Blotting with an anti-λD antibody (Figure 4C) revealed the expected double-band pattern in the fusion phage lanes, with the higher band corresponding to the λD~EGFRvIII fusion protein migrating at approximately 15 kDa and the lower band corresponding to wild-type λD at approximately 11 kDa. Blotting with a rabbit polyclonal anti-EGFRvIII G-version peptide antibody (Figure 4D) detected a strong band at ~15 kDa in the G-version phage lane (lane 1), a weaker band in the Y-version phage lane (lane 2), and no detectable signal in the vector phage lane (lane 3), confirming both the identity of the fusion band and preferential antibody recognition of the displayed antigen.

To evaluate phage-generated antibody binding to the EGFRvIII fusion antigens, the display phage and vector phage samples were probed with sera from IM phage-injected mice (Figure 4E–G). Serum from a mouse injected with G-version display phage produced strong binding to both fusion phage samples (Figure 4E), with a greater signal intensity observed in the G-version phage lane (lane 1) relative to the Y-version phage lane (lane 2). Conversely, serum from a mouse injected with the Y-version display phage demonstrated stronger binding to the Y-version phage than to the G-version phage (Figure 4F). No binding was observed in the vector phage lane in either case; however, a lower set of bands can be seen in the blot probed with the G-peptide serum (Figure 4E), corresponding to the same location of lower wild-type λD bands in Figure 4C. It is not clear whether the lower set of bands in Figure 4E represent the detection of serum anti-λD antibody or anti-EGFRvIII antibody binding to a secondary band of low-migrating EGFRvIII-displayed peptide. Serum from a vector phage-injected mouse produced no detectable signal in the 10–15 kDa region (Figure 4G). Comparable results were observed using sera from the IP study, including samples from both N- and C-terminal fusion phage injection groups (Figure S3).

Collectively, these ELISA and Western blot analyses demonstrate that λ phage-displayed EGFRvIII peptides elicit antigen-specific antibody responses with precise recognition of the homologous peptide sequence and decreased cross-reactivity to closely related variants.

### 3.4. EGFRvIII-Specific Antibody Responses Elicited by Display Phage Injection

After establishing the presence of strong antibody responses against synthetic EGFRvIII peptides, sera from the phage-immunized mice were evaluated for antibody binding to EGFRvIII and wild-type EGFR recombinant extracellular domain proteins. These experiments addressed whether immunization with λD-displayed 13-mer EGFRvIII peptides stimulated antibodies specific to the tumor-associated EGFRvIII receptor while sparing the wild-type EGFR receptor expressed in healthy epithelial tissues [37]. Antibody specificity was assessed by ELISA and Western blotting using sera from both the IP and IM studies.

**Figure 5:**
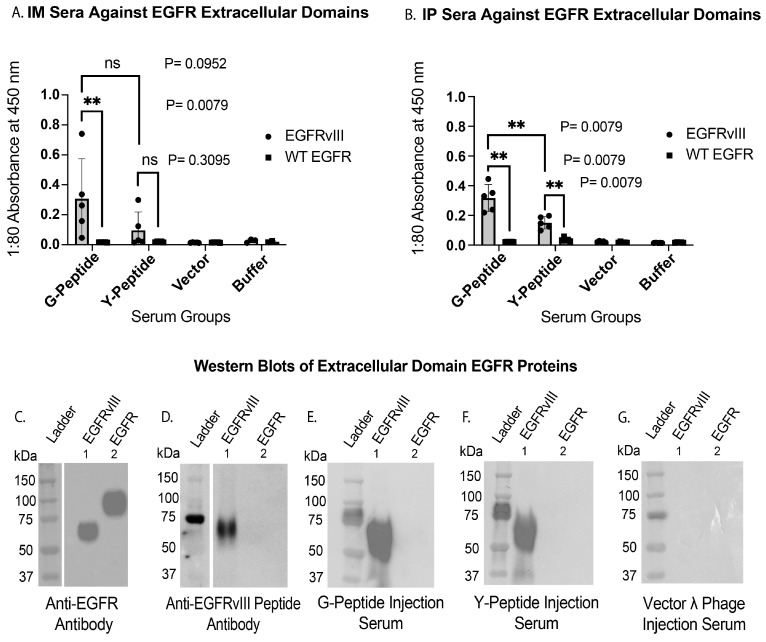
Validation of EGFRvIII-specific immune response by ELISA and Western blot. (**A**,**B**) IM and IP study terminal bleed sera antibody titers were tested by ELISA against EGFRvIII extracellular domain (353 amino acids) and wild-type EGFR extracellular domain (620 amino acids) recombinant proteins. The EGFR coating proteins feature a 10× His-tag on the C terminus for binding to the nickel-coated ELISA plate. EGFR extracellular domains were added to the plate at a 1:1.74 ratio of EGFRvIII to wild-type EGFR to equalize the molecular concentrations of the proteins. The assays utilized anti-mouse secondary antibody. Each data point is an average of two distinct readings per mouse, with five sera averaged per group. The serum groups tested against these proteins are the C-terminal fusions of both the G and Y antigen, vector phage, and buffer. Mouse sera were tested by ELISA in the following serial dilutions: IP = 1:10, 1:20, 1:40, 1:80, and 1:160; IM = 1:80, 1:100, 1:300, and 1:1000. A 1:80 dilution was selected for the subsequent analyses, as the corresponding absorbance values fell within the linear range of the assay without evidence of saturation. Mann–Whitney U test statistical analyses and calculations of standard deviation error bars were performed in Prism 10 software. *p* value asterisks are awarded as follows: ns: *p* > 0.05 (not significant); ** 0.001 < *p* ≤ 0.01 (very significant). (C-G) Western blots, where EGFRvIII extracellular domain and wild-type EGFR extracellular domain proteins were loaded in a 1:1.74 ratio to equalize the molecular concentrations of the proteins. The protein samples were blotted with (**C**) an anti-EGFR antibody (528), (**D**) an anti-EGFRvIII peptide antibody, (**E**) C-terminal G-peptide phage display IM-injected serum, (**F**) C-terminal Y-peptide phage display IM-injected serum, and (**G**) vector λ phage IM-injected serum. Anti-mouse secondary antibody was utilized for all blots, except when using the anti-EGFRvIII peptide antibody (**D**), which required anti-rabbit secondary antibody.

The ELISA analysis of G-peptide injection group sera from the IM study demonstrated distinct binding to the EGFRvIII extracellular domain protein and levels comparable to those of the negative controls against the wild-type EGFR extracellular domain (Figure 5A). The G- and Y-group responses against EGFRvIII were not significantly different through a Mann–Whitney U test, necessitating a larger sample size to confirm the apparent higher G-peptide injection group response. Although individual antibody responses in the IM G-version group exhibited substantial variability (absorbance range 0.04–0.74), the relative difference between the G- and Y-version sera was maintained across all serum dilutions (Figure S4). The signal observed in the Y-peptide IM injection group against EGFRvIII was not found to be significant over the group’s response to EGFR.

In the IP study, sera showed no detectable binding to wild-type EGFR and significant binding to EGFRvIII, particularly in the C-terminal G-version fusion phage group (Figure 5B). In this study, all comparisons were significant, likely due to the closer clustering of data points within groups. The Y group was significantly higher against EGFRvIII than against EGFR, and the G group was shown to be higher than the Y group against EGFRvIII. The higher G- over Y-version binding affinity to EGFRvIII is expected because the EGFRvIII extracellular domain contains the naturally occurring novel glycine at the N-terminal deletion junction.

The closely clustered sera from the IP G-version group (range 0.21–0.44 absorbance) yielded a group-averaged signal similar to that observed in the more varied IM study (mean absorbance of 0.32 for IP and 0.31 for IM). These results support the binding affinity of EGFRvIII~λD fusion phage–elicited antibodies for the mutant receptor across both studies, especially when stimulated by the G-novel junction displayed antigen.

Antibody specificity for the mutant EGFRvIII receptor was further validated by Western blot analysis of recombinant EGFR and EGFRvIII extracellular domain proteins (Figure 5C–G). Blotting with a wild-type EGFR monoclonal antibody (clone 528) detected both EGFRvIII and wild-type EGFR proteins, consistent with a shared domain homology beyond the EGFRvIII-specific N-terminal deletion (Figure 5C). In contrast, a rabbit polyclonal anti-EGFRvIII peptide antibody exclusively bound the EGFRvIII protein and not wild-type EGFR (Figure 5D), establishing a reference pattern for EGFRvIII-specific recognition.

Sera from mice immunized with either the G- or Y-version display phage exhibited strong binding to the EGFRvIII extracellular domain and no detectable binding to wild-type EGFR (Figure 5E,F). Serum from a vector phage-immunized mouse showed no reactivity to either protein (Figure 5G). Equivalent specificity was observed in sera from the IP study across both the N- and C-terminal fusion constructs (Figure S5A–E).

Collectively, these data demonstrate that λD-displayed EGFRvIII peptide immunization stimulates antibodies that selectively recognize the EGFRvIII mutant receptor while exhibiting minimal cross-reactivity with wild-type EGFR. This specificity was consistently observed across independent cohorts through two distinct routes of administration and validated by complementary ELISA and Western blot analyses.

## 4. Discussion

To validate the antibody-stimulating ability of the λD display platform, we tested the following study conditions and display constructions: N-terminal vs. C-terminal antigen fusion, intramuscular vs. intraperitoneal injection route, immunogenicity of EGFRvIII antigen G and Y novel junction amino acids displayed on phage, and the specificity of phage-stimulated antibodies to the EGFRvIII extracellular domain compared to wild-type EGFR. Our findings, confirmed by two live mouse studies, support the further use of the λ phage D protein display system for anti-EGFRvIII immunotherapy and general antibody generation.

The λD display protein protrudes from the phage’s icosahedral head at both N and C termini, allowing for evaluation of antigen efficacy at both fusion locations [46]. Our study found that C-terminal λD antigen fusion stimulated a substantially higher antibody response than N-terminal fusion (Figure 2B). Structural insights from Yang et al. (2000) [53] partially speak to this difference: while both termini protrude from the head, the first 14 N-terminal residues are disordered and converge at the trimer’s three-fold axis. N-terminal fusions may thus disrupt trimerization, leading to either low stability of the virion or the display. Conversely, C terminus residues are fully ordered and protrude at the edge of the trimer structure, creating a more favorable fusion location for particle stability. However, anti-λD antibody Western blots (Figure 2A and Figure S3A) showed comparable incorporation of fusion D proteins in both constructs, indicating that structural differences alone do not account for the immunogenicity gap. It is also possible that exposing the N- or C-terminal end of the EGFRvIII peptide itself affects immunogenicity in the two different constructions. Although the precise cause of low N-terminal immunogenicity remains unclear, the data shown in Figure 2 supports C-terminal fusion as preferable to N-terminal fusion for stimulating an immune response against the EGFRvIII peptide. Future experiments can assess whether low N-terminal immunogenicity is specific to the EGFRvIII peptide or persists with other passenger proteins.

Both IP and IM routes of phage injection produced significant and specific antibody titers by the final bleed day of the two studies; however, the two injection routes demonstrated distinct humoral response patterns (Figure 3). In the IP study, average titers rose immediately and then plateaued while IM study titers gradually increased. The trends of individual mice in the two studies showed several cases of decreasing antibody response in the IP study, while all mice in the IM study demonstrated an increasing antibody trajectory. Additional studies analyzing the antibody response to phage injection over a longer period will be necessary to confirm the significance of the early antibody trends observed here. However, from the data presented in Figure 3, injection of phage via an IM route produced a promising increasing antibody response over time. For the purpose of demonstrating λD display immunogenicity, the similarity in antibody titers observed by the end of two distinct studies suggests that robust antibody response to phage vaccination is reproducible by either injection route.

Fidanza et al. (2021) [44] found that exchanging the EGFRvIII peptide novel glycine residue for tyrosine increased survival in a mouse tumor model treated with the synthetic EGFRvIII peptide conjugated to KLH. For the potential use of both the G and Y novel junction antigens in future phage display cancer models or clinical trials, here we tested both antigen variants displayed on phage for their ability to raise antibodies. Our IM and IP immunization studies found that EGFRvIII peptide-raised antibodies had a higher binding affinity for the homologous peptide than for the alternate peptide variant containing a single amino acid substitution. While both exhibited cross-reactivity, G-antigen-raised antibodies had stronger binding to the G-peptide (Figure 4) and the full EGFRvIII extracellular domain (Figure 5) compared to Y-antigen-raised antibodies. This is unsurprising since EGFRvIII contains the novel glycine matching the G-peptide sequence (but not the Y-peptide). In a therapeutic application, this cross-affinity disadvantage of the Y-version antigen compared to the G-version antigen would need to be weighed against the Y-antigen’s advantage of increased proteasomal cleavage that conferred a survival advantage in Fidanza et al.’s tumor mouse model.

Finally, we demonstrated binding of sera antibodies not only to the synthetic EGFRvIII peptide but also to the full-length EGFRvIII extracellular domain (Figure 5). A crucial consideration of this study was antibody specificity to the target EGFRvIII mutant cancer-variant receptor and not to wild-type EGFR found in healthy cells [36]. We report that antibodies raised by EGFRvIII antigens fused to λ phage particles are specific to EGFRvIII and have minimal cross-reactivity to the wild-type receptor. This specificity for the mutated receptor indicates that antibodies produced by EGFRvIII~λD fusion may be potentially useful against cancer cells displaying EGFRvIII.

## 5. Conclusions

The mouse studies reported here support the use of this λD antigen-display platform to stimulate a rapid and specific antibody response to a passenger peptide antigen. Future experiments in a murine glioma model will be necessary to assess the potential efficacy of the λD~EGFRvIII therapeutic beyond specific antibody generation, such as T cell activation, cytokine stimulation, and evidence of anti-tumor effects. Additionally, it will be important in future studies to compare the λ display system’s immunogenicity to that of a peptide EGFRvIII vaccine, such as rindopepimut. The λ phage-based display system utilized here can be expanded to a multitude of applications. Whether for other cancers with predictable antigen markers or infectious diseases requiring rapid vaccine development, λD display technology demonstrates potential as an immune-stimulating platform.

## 6. Patents

This publication relates to the technology described in the patent publication number 20250082745; title: Bacteriophage Lambda-Vaccine System; published 12, December 2022.

## Figures and Tables

**Table 1:** Bacterial strains utilized in this study to facilitate the generation of EGFRvIII display phage.

Bacterial Strains	Relevant Genetic Markers	Source	Prophage	Strain Use
XTL1212	LE392 pΔ*_BAD_*::*λD* */pLXT42 **	Bush et al. 2026 [3]	_	Strain used as host lawn to titer display phage lysates.
XTL1026	XTL981 Δ*lamB*, Δ*fhuA*/pKM208 ***	Bush et al. 2026 [3]	YXTL1026	Strain used to generate vector control phage lysate.
XTL1059	XTL1026 *tetA*-*sacB*::linker~*D*— The *tetA-sacB* cassette is inserted with linker coding sequence at *λD* gene ATG start.	Bush et al. 2026 [3]	YXTL1059	Parental strain engineered with EGFRvIII peptides at prophage λD N terminus to create display strains MS41 and MS42.
CC001	XTL1026 *D~linker::ccdB-kan*— The *ccdB-kan* cassette is inserted with linker coding sequence at *λD* gene TAA stop.	This study	YCC001	Parental strain engineered with EGFRvIII peptides at prophage λD C terminus to create display strains MS45 and MS48.
MS41	XTL1059—*EGFRvIII* peptide (novel residue G) at prophage *λD* N terminus.	This study	YMS41	Strain used to raise lysate of N-terminal, G-peptide display phage for injection into mice.
MS42	XTL1059—*EGFRvIII* peptide (novel residue Y) at prophage *λD* N terminus.	This study	YMS42	Strain used to raise lysate of N-terminal, Y-peptide display phage for injection into mice.
MS45	CC001—*EGFRvIII* peptide (novel residue G) at prophage *λD* C terminus.	This study	YMS45	Strain used to raise lysate of C-terminal, G-peptide display phage for injection into mice.
MS48	CC001—*EGFRvIII* peptide (novel residue Y) at prophage *λD* C terminus.	This study	YMS48	Strain used to raise lysate of C-terminal, Y-peptide display phage for injection into mice.

* indicates that the gene preceding the double colon is replaced by the gene following it. **: pLXT42 is a plasmid that expresses wild-type λD under the tunable pBAD promoter, inducible by arabinose. It also contains the following elements: *AmpR* gene and *pMBori.* ***: pKM208 is a pSC101-based plasmid that expresses Red functions *exo bet* and *gam* from the *lac* promoter for recombineering. Obtained from Ken Murphy. ~ symbol refers to a genetic fusion. Y = temperature-sensitive *cI857* mutation.

**Table 2:** Prophage strains complementary to *E. coli* strains in Table 1 with listed genetic modifications.

Prophage Name	Prophage Genotype	Source	Prophage Use
YXTL1026	λ*cI857*, *Sam7*, Δ*b2*, *E_E158K_*	Bush et al. 2026 [3]	Vector phage control for mouse studies.
YXTL1059	YXTL1026 *tetA*-*sacB*::linker~*D*— The *tetA-sacB* cassette is inserted with linker coding sequence at *λD* gene ATG start.	Bush et al. 2026 [3]	Parental prophage recombineered with display peptide at λD N terminus.
YCC001	YXTL1026 *D~linker::ccdB-kan*— The *ccdB-kan* cassette is inserted with linker coding sequence at *λD* gene TAA stop.	This study	Parental prophage recombineered with display peptide at λD C terminus.
YMS41	XTL1059— *EGFRvIII* peptide (novel residue G)~linker~*D,* linker and EGFRvIII antigen are inserted at *λD* gene ATG start.	This study	Prophage induced to create lysate of N-terminal, G-peptide phage for mouse injection.
YMS42	XTL1059— *EGFRvIII* peptide (novel residue Y)~linker~*D,* linker and EGFRvIII antigen are inserted at *λD* gene ATG start.	This study	Prophage induced to create lysate of N-terminal, Y-peptide phage for mouse injection.
YMS45	CC001— *D~linker~EGFRvIII* peptide (novel residue G), linker and EGFRvIII antigen are inserted at *λD* gene TAA stop.	This study	Prophage induced to create lysate of C-terminal, G-peptide phage for mouse injection.
YMS48	CC001— *D~linker~EGFRvIII* peptide (novel residue Y), linker and EGFRvIII antigen are inserted at *λD* gene TAA stop.	This study	Prophage induced to create lysate of C-terminal, Y-peptide phage for mouse injection.

~ symbol refers to a protein fusion. Y = temperature-sensitive *cI857* mutation.

## Data Availability

The original contributions presented in this study are included in the article/Supplementary Material. Further inquiries can be directed to the corresponding authors.

## Supplementary Materials

The following supporting information can be downloaded at: https://www.mdpi.com/article/10.3390/vaccines14030282/s1, Figure S1: Validation of C-terminal display efficacy by analysis of IP study sera full dilution series and full study bleed schedule; Figure S2: IP and IM study sera tested on ELISA against G and Y synthetic 13-mer EGFRvIII peptides; Figure S3: IP study complementary display phage western blots of 10–15 kDa λD region blots shown in Figure 4C–G; Figure S4: ELISA analysis of IM sera in dilution against EGFRvIII and EGFR extracellular domains; Figure S5: IP study complementary western blots to those shown in Figure 5C–G; Figure S6: IM study full phage sample mouse serum blots; Figure S7: Western blot and Colloidal Blue stain SDS-PAGE gel comparison.
