# Supplementary material for: Lambda Phage-Based Antibody-Stimulating Platform Targeting EGFRvIII
Source: Vaccines (Basel). 2026 Mar 23;14(3):282. doi: 10.3390/vaccines14030282 (PMC13030582; doi:10.3390/vaccines14030282)
Supplement: Supplementary file 1 [file vaccines-14-00282-s001.zip › vaccines-4153492-supplementary.pdf]

vaccines

MDPI

Supplemental Materials

Supplemental S.1. Additional ELISA and western blots from IP and IM studies

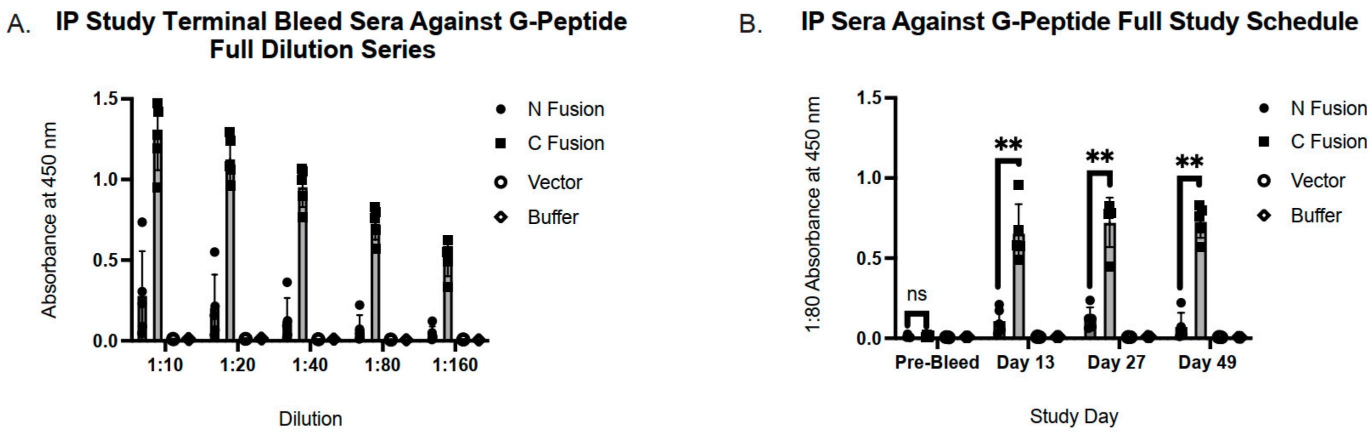

Figure S1. Validation of C-terminal display efficacy by analysis of IP study sera full dilution series and full study bleed schedule. Each data point is an average of two distinct readings per mouse, with five sera averaged per fusion group. Mann-Whitney U test statistical analysis and calculation of standard deviation error bars were performed in Prism 10 software. P value asterisks are awarded as follows: ns = $p > 0.05$ (not significant); * = $0.01 < p \leq 0.05$ (signifi-cant); ** = $0.001 < p \leq 0.01$ (very significant). Anti-mouse secondary antibody was utilized in these assays. (**A**). ELISA absorbance of terminal bleed IP study mouse sera antibody binding to the G-version synthetic peptide in 2-fold serial dilutions (1:10, 1:20, 1:40, 1:80, and 1:160). P values comparing N and C fusion bars = 0.0079 (**) for all dilutions. (**B**). ELISA antibody absorbance against the G-version synthetic peptide of IP study sera at a 1:80 dilution from every serum collection timepoint throughout the study. P values = 0.8413 (Pre-Bleed), 0.0079 (Day 13), 0.0079 (Day 27), and 0.0079 (Day 49). Figure included to demonstrate consistent low N-terminal antibody absorbance compared to C-terminal display injection sera over the course of the IP study and in mutliple dilutions.

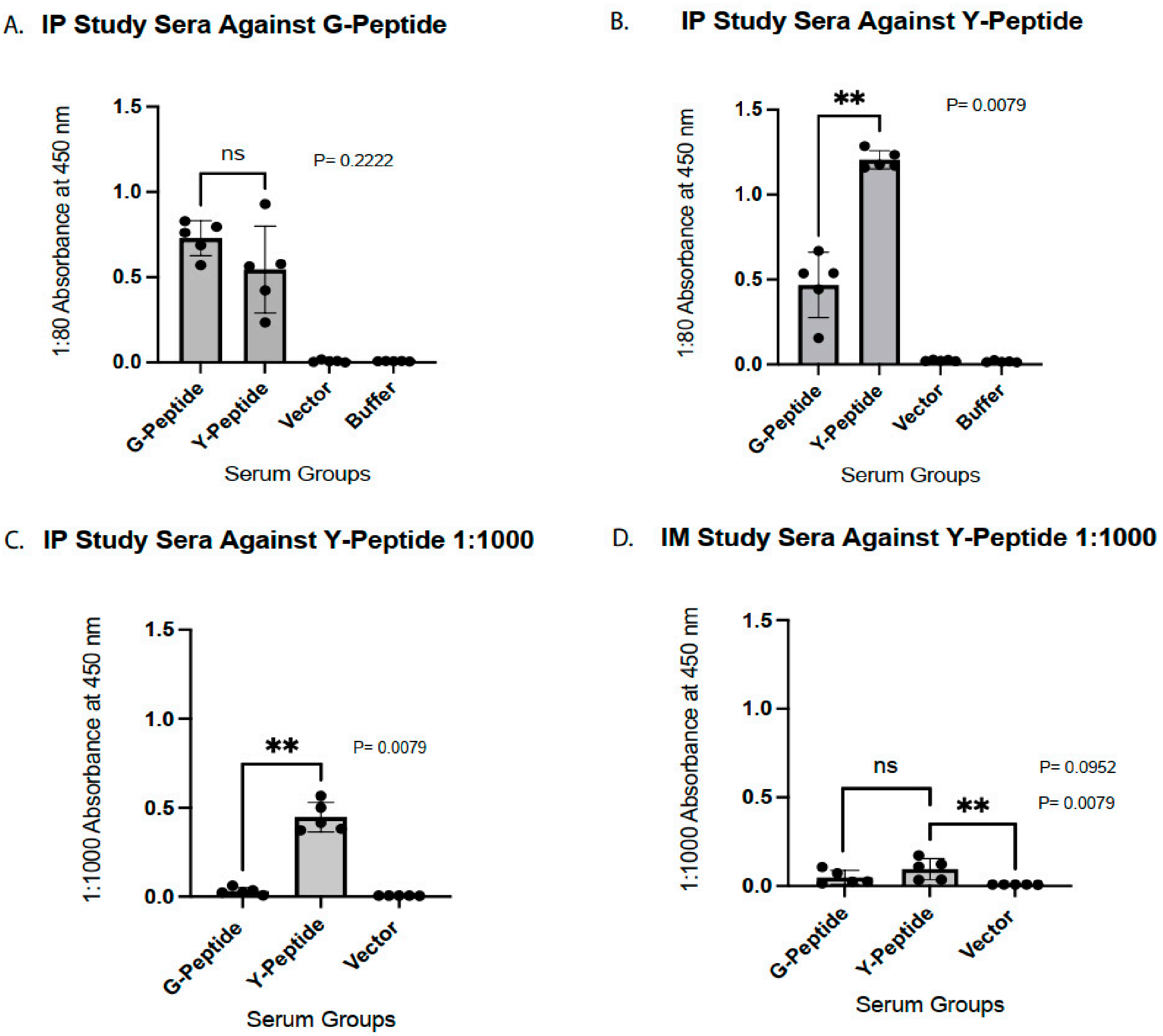

Figure S2. IP and IM study sera tested on ELISA against G and Y synthetic 13-mer EGFRvIII peptides. Each data point is an average of two distinct readings per mouse, with five sera averaged per fusion group. Mann-Whitney U test statistical analysis and calculation of standard deviation error bars were performed in Prism 10 software. P value asterisks are awarded as follows: ns = $p > 0.05$ (not significant); * = $0.01 < p \leq 0.05$ (signifi-cant); ** = $0.001 < p \leq 0.01$ (very significant). In the IP study, mouse sera were tested by ELISA in 2-fold serial dilutions (1:10, 1:20, 1:40, 1:80, and 1:160). A 1:80 dilution was selected for analyses, as the corresponding absorbance values fell within the linear range of the assay without evidence of saturation for sera tested against the G-peptide. Sera were retested in 10-fold dilutions against the Y-peptide to obtain values in a linear range at a 1:1000 dilution, as they were still oversaturated at a 1:80 dilution. Anti-mouse secondary antibody was utilized in these assays. (**A**). IP study sera against the EGFRvIII G-antigen 13-mer peptide, showing G-peptide injections with a higher response than Y-peptide injections, though the difference was not found to be significant. (**B**). IP study sera against the EGFRvIII Y-antigen 13-mer peptide, showing Y-peptide injection sera with a significantly higher response than G-peptide injection sera. (**C**). IP study sera against the EGFRvIII Y-antigen 13-mer peptide at 1:1000 dilution, showing Y-peptide injection sera with high absorbance, indicating saturation at a 1:80 dilution in S2B. (**D**). IM study sera against the EGFRvIII Y-antigen 13-mer peptide at 1:1000 dilution, showing Y-peptide sera absorbance only slightly elevated and non-significant over G-peptide sera. However, Y-peptide sera

absorbance was significantly higher than the vector group. Overall findings: IP study Y-peptide serum against the Y-peptide was over-saturated at a 1:80 dilution, necessitating further dilution to accurately capture the higher response of antibody binding to the corresponding Y-peptide. The titer of Y-serum against the Y-peptide was lower in the IM study compared to the IP study.

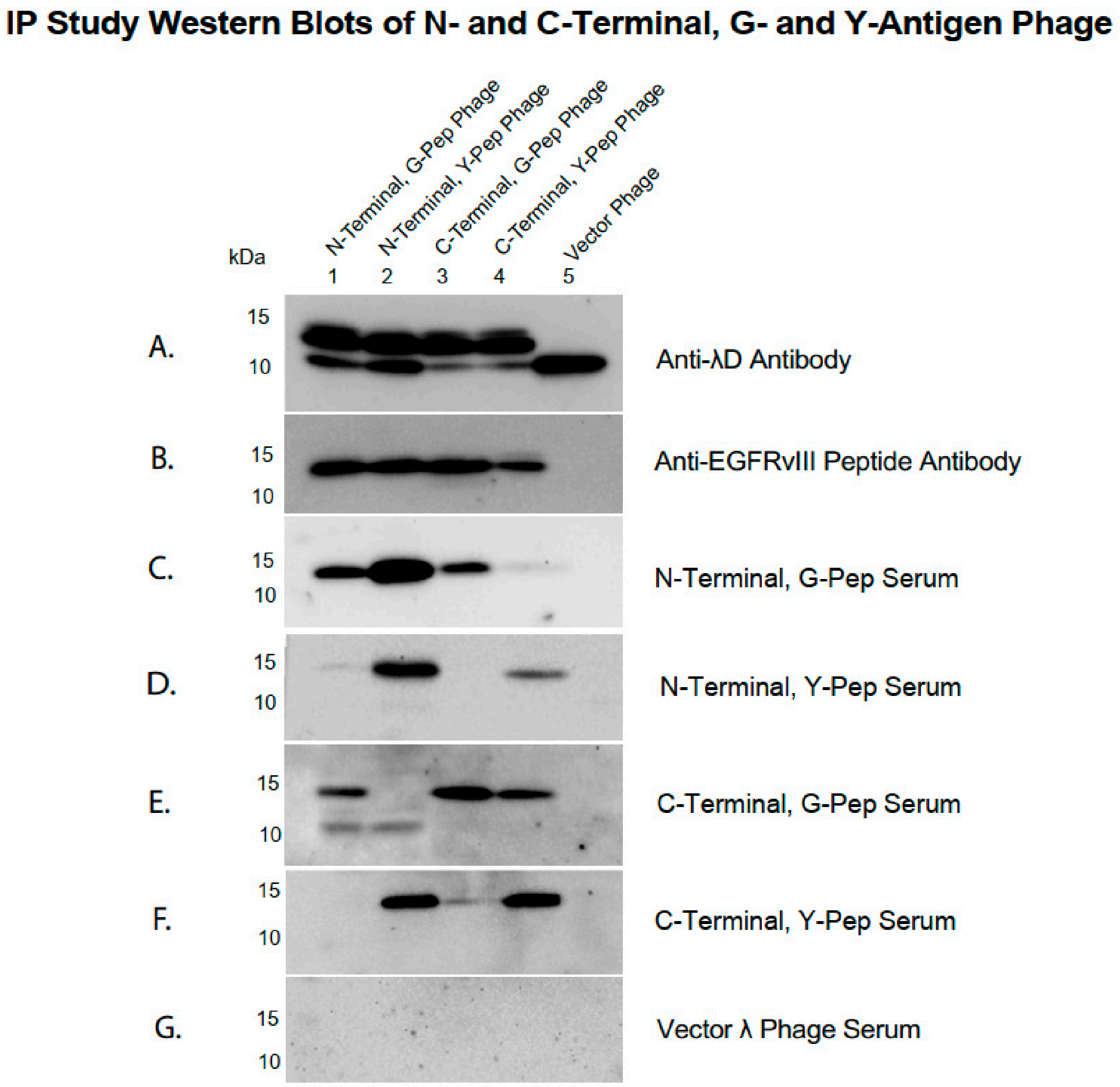

Figure S3. IP study complementary display phage western blots of 10-15 kDa λD region blots shown in Figure 4C-G. Phage blotted against N-terminal and C-terminal injection group sera from the IP study. N-terminal fusion phage with the G-peptide antigen (lane 1), N-terminal fusion phage with the Y-peptide antigen (lane 2), C-terminal fusion phage with the G-peptide antigen (lane 3), C-terminal fusion phage with the Y-peptide antigen (lane 4), and vector phage (lane 5) were loaded onto a gel. $5x10^9$ pfu/ml phage added to each lane for A and B. $2x10^{10}$ pfu/ml phage added to each lane for C-G. Phage sample nitrocellulose membranes were blotted with an anti-λD antibody (**A**), an anti-EGFRvIII peptide antibody (Y-peptide phage titers increased 10-fold to visualize band) (**B**), N-terminal G-peptide fusion phage serum (**C**), N-terminal Y-peptide fusion phage serum (**D**), C-terminal G-peptide fusion phage serum (**E**), C-terminal Y-peptide fusion phage serum (**F**), and vector λ phage serum (**G**). All blots were blotted with anti-mouse secondary antibody, except when using the Anti-EGFRvIII Peptide Antibody (S3B) which required anti-rabbit secondary antibody. Blots show preferential serum antibody affinity for the displayed peptide antigen version the mouse received by injection.

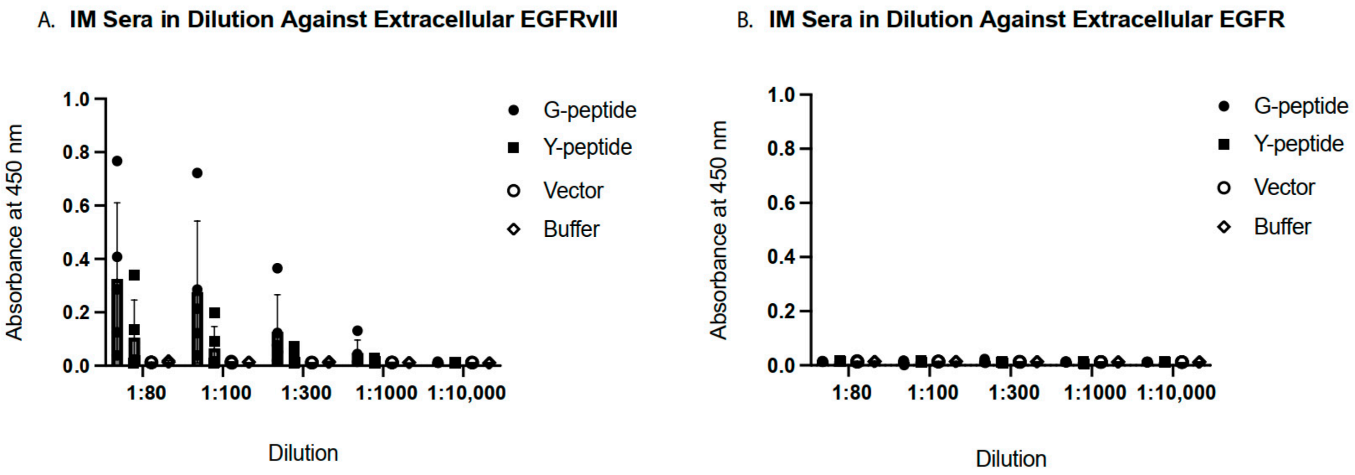

Figure S4. ELISA analysis of IM sera in dilution against EGFRvIII and EGFR extracellular domains. IM study sera tested through ELISA for antibody production against EGFRvIII and EGFR extracellular domain proteins. Each mouse serum was tested twice and averaged in five dilutions: 1:80, 1:100, 1:300, 1:1000, 1:10000. Each bar is an average of five mice. Calculation of standard deviation error bars was performed in Prism 10 software. Anti-mouse secondary antibody was utilized in these assays. (**A**). IM sera against EGFRvIII extracellular domain. These data demonstrate a consistent pattern of high G-antigen group antibody response compared to the Y-antigen group response in several dilutions. Data was included to demonstrate consistency of comparative signal across many dilutions. (**B**). Mouse sera samples tested against EGFR extracellular domain in the same dilutions as A. These data demonstrate a lack of response against the wild-type EGFR protein.

IP Study Western Blots of Extracellular Domain EGFR Proteins

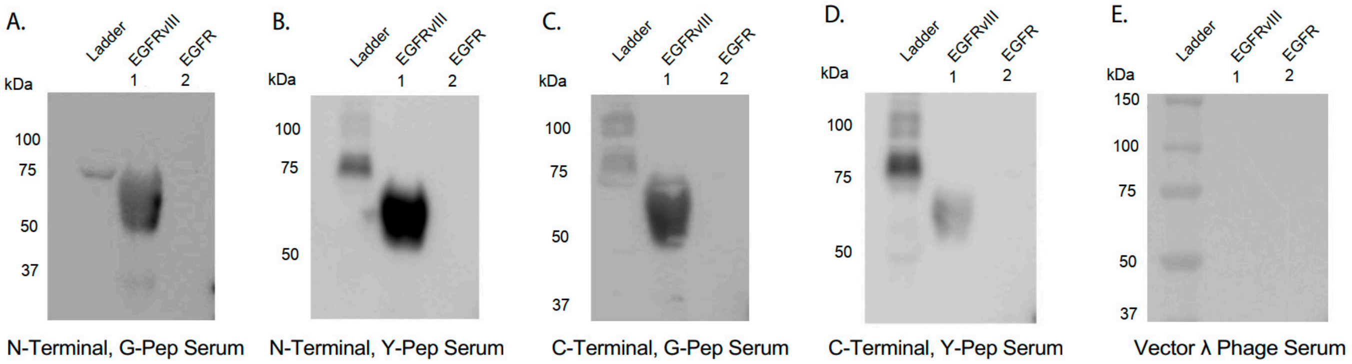

Figure S5. IP study complementary western blots to those shown in Fig. 5C-G. IP injection-derived mouse sera selectively recognizes the EGFRvIII extracellular domain. EGFRvIII extracellular domain protein and wild-type EGFR extracellular domain proteins were loaded in a 1:1.74 ratio to equalize the molecular concentrations of the proteins. Protein samples were blotted with (**A**). N-terminal G-peptide fusion phage-injection serum, (**B**). N-terminal Y-peptide fusion phage-injection serum, (**C**). C-terminal G-peptide fusion phage-injection serum, (**D**). C-terminal Y-peptide fusion phage-injection serum, and (**E**). Vector λ phage-injection serum. All blots were blotted with anti-mouse secondary antibody. Blots show mouse serum antibody binding only to EGFRvIII extracellular domain (lane 1) and no binding to wild-type EGFR protein (lane 2). The leftmost lane is the molecular marker.

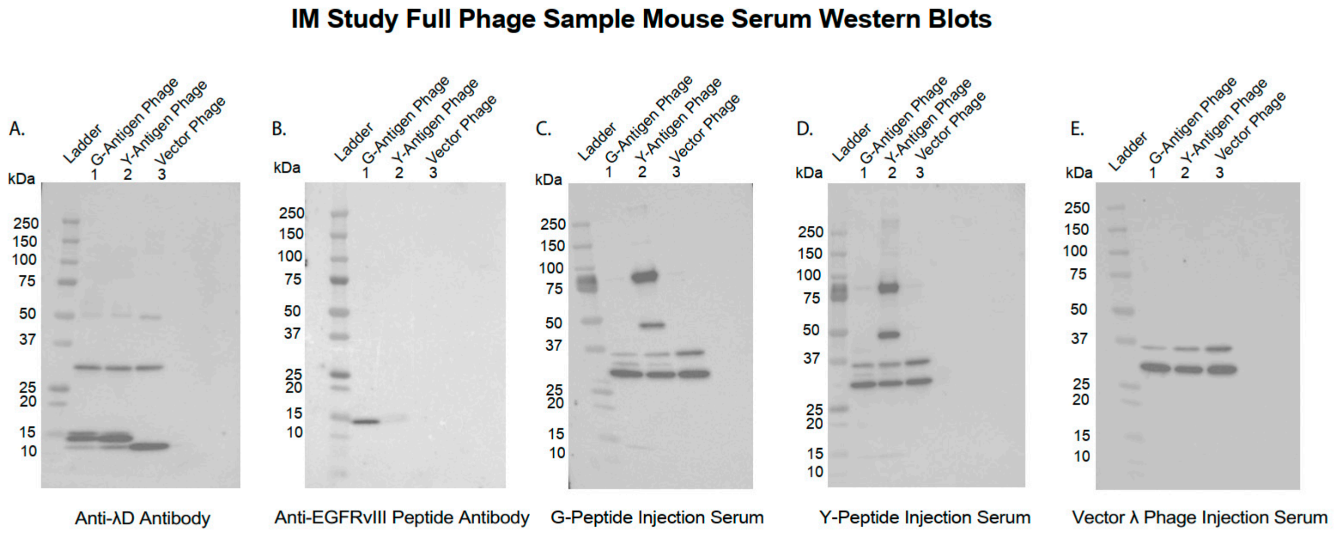

Figure S6. IM study full phage sample mouse serum blots. Full molecular weight range western blots expanding on the 10-15 kDa range shown in Fig. 4C-G. C-terminal fusion phage with the G-peptide antigen (lane 1), Y-peptide antigen (lane 2), and vector phage (lane 3) blotted with an anti-λD antibody (**A**), and anti-EGFRvIII peptide antibody (**B**), C-terminal G-peptide fusion phage serum (**C**), C-terminal Y-peptide fusion phage serum (**D**), and vector λ phage serum (**E**). All blots were blocked with anti-mouse secondary antibody, except when using the Anti-EGFRvIII Peptide Antibody (S6B) which required anti-rabbit secondary antibody. Bands between 25-37 kDa correspond to λ phage proteins V and E, as identified through in-gel digestion protein identification (data not shown). The full mouse serum blots shown here in Figure 6 were cut at 20 kDa after the full blots were imaged and re-imaged (shown in Figure 4) to capture the 10-15 kDa bands clearly without interference from stronger bands above 20 kDa.

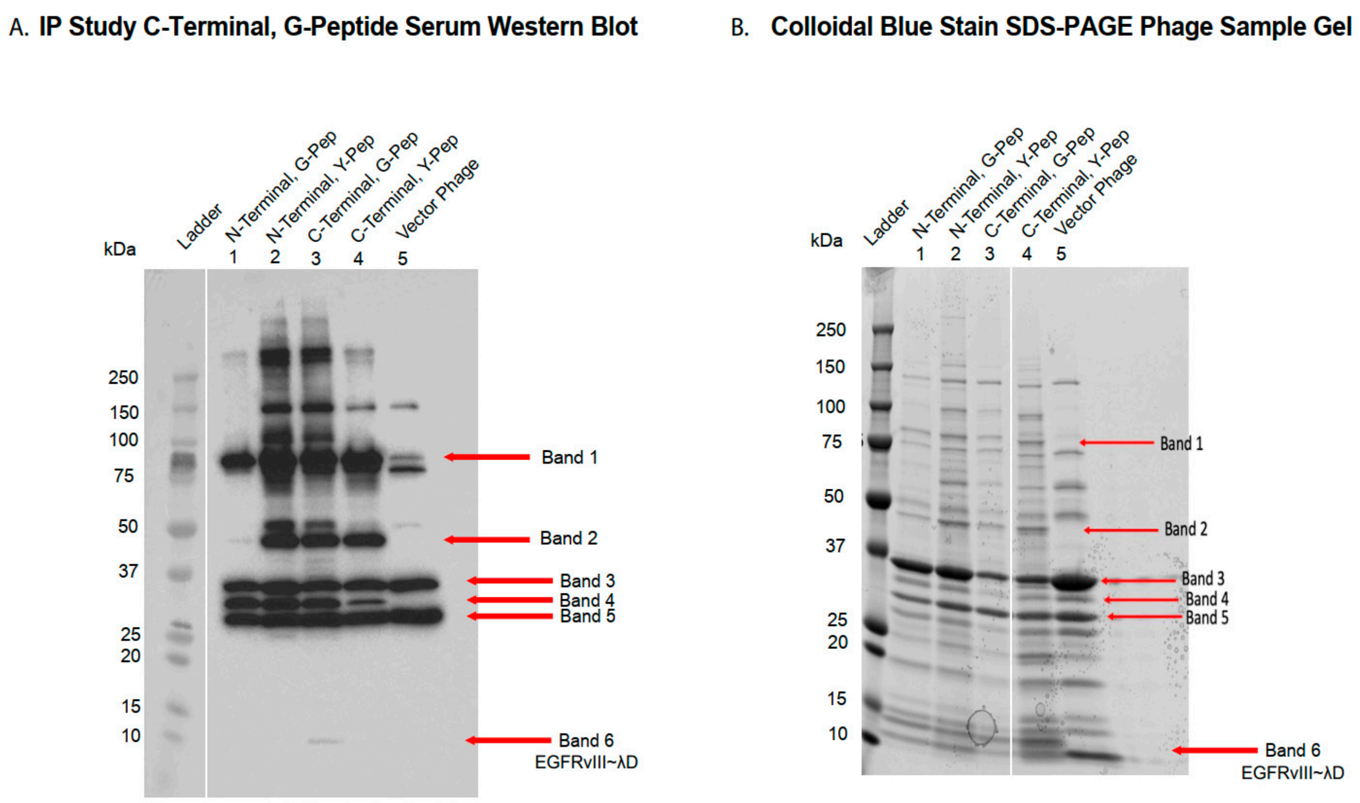

Figure S7. Western blot and Colloidal Blue stain SDS-PAGE gel comparison. 5x10[10] pfu/ml phage added to each lane for A and B. (**A**). Western blot of C- and N-terminal display phage as well as vector phage run on an SDS-PAGE gel, transferred to a nitrocellulose membrane, and blotted with C-terminal, G-peptide injection mouse serum from the IP study. Membrane was blotted with anti-mouse secondary antibody and imaged with chemiluminescence. (**B**). Phage samples were run on SDS-PAGE gel and stained with Coomassie Brilliant Blue G-250 dye to visualize the full protein profile of phage samples. Emphasized bands (1-6) are those that correspond to Fig. S7A major bands as well as bands seen in IM study serum western blots in Fig. S6C and S6D.
